# Social Media Use in E-Learning amid COVID 19 Pandemic: Indian Students’ Perspective

**DOI:** 10.3390/ijerph19095380

**Published:** 2022-04-28

**Authors:** Abu Elnasr E. Sobaih, Ishfaq Ahmad Palla, Abdul Baquee

**Affiliations:** 1 Management Department, College of Business Administration, King Faisal University, Al-Ahsaa 31982, Saudi Arabia; 2 Faculty of Tourism and Hotel Management, Helwan University, Cairo 12612, Egypt; 3 Library and Information Science Department, School of Media and Communication, Pondicherry University, Pondicherry 605014, India; ishfaqpalla63@gmail.com (I.A.P.); imabaquee@gmail.com (A.B.)

**Keywords:** social media, student engagement, student performance, e-learning, COVID-19, higher education institutions, India

## Abstract

As a result of the COVID-19 epidemic, most educational institutions shifted to online education. Students and faculty members in many public institutions, particularly those in developing countries, are hampered by the absence of formal online learning management systems. Responding to COVID-19, many institutions in developing countries adopted social media sites to maintain e-learning and sustain education process. The distinction between online and real-world communities is becoming increasingly narrow, especially among the younger generations who have grown up with social media at their fingertips. This research explores perspectives of higher education students in India regarding the use of social media for e-learning amid the COVID-19 pandemic. For this purpose, an online questionnaire was directed to a sample of higher education students in India via a personal network. The results showed that students were more satisfied with their use of social media because of their perceptions of its ease of use and usefulness. The majority of the students are active on social media for 1–2 h daily (*p* < 0.01). YouTube was the platform of choice among all the respondents (n = 154; 36%). The results confirmed that students feel that social media websites have a significant positive impact on their overall academic performance (*p* < 0.01). Novel methods of teaching and learning are constantly being sought out by educators. The present moment is an opportunity to examine and analyze the theoretical benefits of social media technologies and consider their relative advantages for education through the use of technology’s ability to enhance student learning.

## 1. Introduction

The recent COVID-19 outbreak is posing a significant challenge to educational institutions. The COVID-19 outbreak has thrown the global economy into disarray. Higher education institutions have switched from face-to-face teaching to online teaching. As a result of the pandemic, the higher education system has moved online in most countries, reflecting a need for more training for educators in digital technology, especially in countries and institutions that are adopting e-learning for the first time. Xie et al. [1] points out that this unexpected epidemic forced institutions globally to shift from traditional and conventional classrooms to online classrooms. There is also an argument that this adoption of online learning will continue to persist partially post-pandemic with conventional learning [1]. Nonetheless, many public institutions, particularly those in developing countries, such as India, lack formal online learning management systems. Like governments in many countries, the government of India has mandated that higher education students be taught online or via virtual classrooms rather than face-to-face. Those accustomed to teaching in real-time classrooms will need to learn how to make use of asynchronous learning. Asynchronous learning allows educators to prepare lesson plans with greater freedom, while also allowing students to review these lessons several times at their convenience [2]. This type of learning is most effective when delivered digitally through a proper leaning management system. For students, educators do not necessarily need to deliver material at a specific time; they can access it online and engage with it at their own convenience. Educators can monitor student participation and set up online appointments for students who have specific questions or needs. Asynchronous digital classrooms allow educators and students to have more time to think and collaborate [3]. This is thanks to technological developments in portable devices, with multimedia capabilities that facilitate e-learning for students. This generation of college and university students relies heavily on social media to stay in touch with friends, parents and educators [4,5]. It is not uncommon for students to use their smartphones, tablet computers, laptops and many other electronic devices every single day.

A review of research on the use of social media for e-learning, particularly amid the COVID-19 epidemic (see, for example, [6,7,8]), showed a limited understanding on how digital technologies, such as virtual or online learning situations and social media, can help students achieve their educational goals. In spite of the fact that social media can be used as an educational tool, little is known about “how it affects students’ motivation in e-classroom as well as their academic performance”. The present study sought to answer the above research questions. More specifically, this research study aims to assess students’ choices and motivational factors behind the use of social media; to examine the impact on academic success/grades; to assess attitudes and beliefs towards social media educational contexts during the COVID-19 pandemic.

## 2. E-Learning amid the COVID-19 Pandemic

An e-learning program is a traditional curriculum supplemented by digital resources. E-learning can take place inside or outside a traditional classroom due to the widespread use of computers and the internet [9]. There is a growing interest among academics and policymakers alike in e-learning, which is reshaping the educational landscape. Online learning activities such as posting to discussion forums and looking up information can provide students with many practical online skills in a more incidental and informal manner. Learners could benefit from these skills, which include critical analysis of resources, effective online communication, and the ability to decipher and filter data [10]. As a result of the COVID-19 pandemic, e-learning has replaced traditional teaching methods. The COVID-19 pandemic posed a threat to humanity, as it forced the shutdown of a wide range of global activities, including educational ones in many countries, including India. Before the current pandemic, e-learning was not widely accepted as a legitimate form of education or learning in many developing countries, such as India. This is especially true for many public universities in developing countries, which have no access to formal learning management systems. In order to stop the spread of the virus, higher educational institutions have been forced to switch to e-learning using the educational platforms available. Since global restrictions on the spread of COVID-19 were implemented, higher educational institutions have begun providing the majority of their services via the internet, including lectures and various assessments, via multiple platforms, for over 60% of students around the world [11]. Students’ willingness to use e-learning systems in developed countries is less of an issue than it is in developing countries, because significant progress has already been made in the adoption and use of e-learning systems by students. It is noted that the digital divide between developed and developing countries remains a major obstacle to the adoption of e-learning systems [12,13]. Due to the COVID-19 lockdown, many universities have had to implement online teaching and learning. However, most universities and colleges have found the transition from classroom to online learning difficult, despite some notable successes. The transition was more difficult in many developing countries, especially with the absence of formal learning management systems and limited digital resources [7,8].

## 3. The Use of Social Media for E-Learning

Social media platforms are among the simplest and most effective means of disseminating information [14]. Over the past decade, social media has become the primary method of mass digital communication across different organizations. The ability to connect with individuals who share similar values, interests, or aspirations is becoming increasingly commonplace because of the internet’s ability to facilitate networking. Since students are responsible for their own education in online environments, self-regulation of learning is important for dealing with e-learning [15]. Nevertheless, in the last few years, the world has seen a rise in social media websites, which are “becoming increasingly pervasive in higher education” [16] and have established a new phenomenon on the internet. Students’ personal lives have grown increasingly intertwined with their academic pursuits because of remarkable technical breakthroughs and the rapid expansion of social media websites [17]. With the advent of new educational technologies, such as social media, educators can better engage students both inside and outside of the classroom, ultimately affecting their academic performance [18]. However, social media can be a boon for one reason and bane for another, and the educational benefits of incorporating social media into learning situations are debatable. A number of research works [19,20,21] in the field have highlighted that the use of social media websites can have a beneficial or negative impact on students’ academic performance. Self-motivation is fostered by the informal and autonomous nature of social media [19]. In light of the rapid proliferation of social media platforms in educational systems, scholars and educators have been compelled to examine how these platforms have impacted their education. Many university students and faculty members use social media sites such as Facebook and Twitter to share ideas and resources for classroom instruction [20]. The popularity and frequency with which students use social media shows that, when used effectively, these networks may encourage out-of-class participation, which may ultimately boost academic success [20]. In light of these findings, it appears that students’ sense of community in the classroom is intertwined with other aspects of the classroom. Empirical evidence in the field of online education also supports this line of thinking [21].

The negative facets of social media websites have also been elaborately discussed. Using social media in educational settings has come under fire from researchers for its potential impacts on academic performance [22,23,24,25,26,27]. Roux and Parry [28] emphasized that time spent on social media can be troublesome and has a detrimental impact on academic achievement, because students spend very little time socializing face to face or in person with other people as they spend more time on social media, and this reduces their communication abilities. Some studies [29,30] concentrated on the impact of social media on Pakistani students’ education and on their lifestyles. The study indicated that teenagers’ and children’s usage of social media could damage their lives and have a detrimental impact on their education [30]. Students’ college grades suffered as a result of their excessive use of Facebook. Students’ use of Facebook while carrying out schoolwork was found to have a negative impact on their grade average. Researchers found that extracurricular social media use by students, particularly weaker ones, was detrimental to academic performance [25,31]. It is difficult for many educators to implement social media in education because of privacy concerns [4]. Using social media as a teaching tool is more difficult for educators because they must uphold a duty to protect students’ privacy [32,33]. Mental health is harmed among pupils who are addicted to social media [34]; they are becoming increasingly depressed and engaging in self-harming behavior because of excessive use of both their smartphones and social media [35].

## 4. Methods

### 4.1. Population and Sample

The research population of this study included students in public higher education institutions in India. According to Krejcie and Morgan [36], the sample size for a population upwards of 1 billion should be 384 or more. Our targeted sample gave a total of 500 responses. This number of questionnaires is inconsistent with previous studies on social media [7,8]. Students were accessed through personal networks, e.g., through the teachers of the students. A total of 431 questionnaires were valid for analysis, with a response rate of 86%. We conducted a nationwide survey and distributed questionnaires to students in different institutions across the country in order to achieve our study objectives. Participants consisted of undergraduate and postgraduate students from various Indian public universities.

### 4.2. Data Collection 

The best practices for online surveys, suggested by Evans and Mathur [37], were adopted for ensuring proper data collection. The questionnaire forms were sent via email, WhatsApp and other social media platforms to students in different higher education institutions in India. The adopted best practices included giving a brief message for students about the objectives of the study and their participation for research purposes. There were some simple instructions on how they should fill in the questionnaire form. We adopted an easy design of questionnaire, relying on option selection rather than writing. We also used pretested research items (more information in Section 4.3). We accessed students through our personal networks in different higher education institutions. We covered as wide a geographical area as possible to obtain a good representation of the population. The research team members followed up with the teachers and their students to answer any questions and ensure quick and appropriate responses, justifying our excellent response rate. The data of this study were collected during the first semester of the academic year 2021–2022, primarily during December 2021.

### 4.3. The Research Instrument

The questionnaire was divided into three main sections. Participants’ gender, education details and amount of time spent on social media were included in Section A of the questionnaire. Section B included social media platforms of choice, purpose of using social media and motivation associated with social media. Section C included social media’s impact on their overall academic achievement and their attitudes toward and beliefs about social media use in educational contexts. The full items of the questionnaire form are presented in Appendix A. Various rating scales were developed to directly measure a participant’s emotions. The Likert scale is the most commonly employed rating scale. Likert scales can be used in order to determine how people feel about an object, an idea or a phenomenon. Attitudes toward and beliefs about social media use in educational contexts were measured using a five-point Likert scale. We used previously validated survey items to measure the constructs and contextualized them to fit our research methodology. Items examined students’ attitudes and motivations towards the use of social media for learning purposes and students’ academic performance [38,39].

### 4.4. Data Analysis

The Statistical Package for the Social Sciences (SPSS) was used for analyzing the collected data. Numbers and percentages were used for descriptive results, especially for the profiles of the respondents. A *p*-value (<0.01) was deemed significant for the chi-square test, which was employed to look for a link between variables. 

## 5. Key Findings

Out of the total respondents (n = 431), 313 were male (73%) and the remaining 118 respondents were female (27%). The majority of respondents were postgraduate (65%) compared with 35% undergraduate students. Respondents in different disciplines or sciences are presented in this study (Table 1). As far as time spent on social media on a daily basis is concerned, it was found that 50% (n = 215) of respondents spent 1–2 h on social media daily, whereas 30% (n = 130) respondents spent 3–4 h on social media in a day. Additionally, only 10% of the respondents spent less than 1 h daily on social media and another 10% spent more than 5 h on social media daily (Table 1).

Table 2 presents the social media platforms of choice. It is revealed that the largest share of the male respondents (n = 107; 34%) chose YouTube as their preferred social media platform, followed by Facebook (n = 89; 28%), Twitter (n = 51; 16%) and ResearchGate (n = 34; 10%). Similarly, among the female respondents, YouTube has the highest percentage of users. However, more female respondents preferred ResearchGate over Twitter (see Table 2).

Table 3 highlights the factors which motivated the respondents to use social media sites. Nearly half of the total male respondents (n = 146; 47%) used social media for their personal purposes, i.e., communication with their friends and families, but of the female respondents, a larger number of students (n = 44; 37%) used social media for entertainment. There were no major differences between male and female usage of social media for information-seeking purposes: 63 male respondents (20%) compared to 23 female students (19.5%)**.** The other purpose for using social media among both male and female respondents was altruism (see Table 3).

Table 4 shows an interesting finding that 77% male students (n = 240) answered that social media helped them to improve their marks or grade during the COVID-19 pandemic, and 60% female students gave this answer. This reflects the value of social media usage for educational purposes. 

Table 5 represents the impact of social media on overall academic performance. Here, our study found that 63% male respondents (n = 197) agreed that social media helped them in improving their overall academic performance, and 25% (n = 79) male students said that social media has a moderate impact on overall academic performance. In terms of female students, less than 50% students (n = 53) went with the option that social media has a highly positive impact on their overall academic performance. Of the female students, 33% (n = 39) said social media has a highly negative impact on their overall academic performance, while 11% students from the same category claimed a negative impact of social media on their overall academic performance.

Overall, the use of social media sites for education shows positive attitudes and beliefs among the respondents. The majority of them (above 80%) agreed or strongly agreed that they enjoy using social media as it provides better learning opportunities, improves students’ outcomes and makes learning interactive. Meanwhile, less than 15% responded that the use of social media causes distraction from their online study during the pandemic (Table 6).

## 6. Discussion

Information and communication technologies (ICTs) are becoming increasingly important in the teaching and learning process due to their rapid spread throughout society and their contribution to the education process [40]. As the need for educational resources grows, new and exciting ways to address those needs have emerged, thanks in part to the advancements made possible by modern technology. Educators and institutions are constantly experimenting with social media tools in the hope of involving and engaging students, and enhancing the learning community among their students. Social media has the potential to transform the traditional lecture classroom, opening new avenues of communication and learning that lecture classes alone are unable to provide [41]. Students’ learning can be aided by fostering an online learning community [42,43]. Higher education institutions are always looking for ways to increase student learning through the use of ICT tools [42,44]. As discussed earlier in the literature review, in higher education, the usage of social media can benefit students in a variety of ways, including deeper comprehension and learning, improved recall, increased involvement and engagement, more structure and attention, and improved teamwork and organization [4,5,6,7,8]. It is widely accepted that the use of Web 2.0 resources, such as YouTube, Facebook and ResearchGate, etc., enhances teaching and learning processes and is “considered as a necessity for the development of lifelong learners” [45,46,47]. This notion is supplemented by the increase in the number of students who use various social media platforms to communicate for scholarly purposes.

Our results suggest that use of social media has led to improvements in the grades of both male and female respondents (males: n = 240, 77%; females: n = 71, 60%). Participants were also asked about the impact of using social media. Male (n = 197; 63%) and female (n = 53; 45%) respondents highlighted that the use of social media had a highly positive impact on their overall performance (*p* < 0.01). These findings are in line with some studies in the literature, e.g., Ref. [6], showing that the use of social media for e-learning supports grades and academic performance, especially amid the COVID-19 pandemic. The results are inconsistent with other studies, e.g., Ref. [19], which claim that social media usage does not support students’ grades.

In terms of the time spent on different social media platforms, half of respondents (n = 215; 50%) used social media for 1–2 h daily. We found that students rely heavily on YouTube (n = 154; 36%). It could be because watching educational content on YouTube makes learning more enjoyable and interactive and boosts the motivation of students to learn. Various studies have found a positive effect of YouTube on students [48,49,50,51]. Aside from the obvious convenience of being able to access course materials via social media and mobile devices, there are some other advantages to this method of learning. Social media and mobile devices are widely regarded by students as inexpensive and convenient sources of relevant information [52]. Other social media platforms, such as Twitter and Facebook, are linked to higher levels of student involvement and information sharing [53]. In addition to supporting student education, Web 2.0 tools can also help individuals overcome communication and writing difficulties. Social media can be used beyond education.

Our results highlighted that the majority of the students use social media sites for personal use (n = 184; 43%), followed by entertainment (n = 134; 31%). Web 2.0 tools can be useful, but excessive use can lead to a lack of focus on learning and a decrease in academic performance, as highlighted by [13,54,55]. Many educational institutions are discovering the benefits of social media’s rapid rise in India, especially amid the COVID-19 pandemic. Social media can have a positive impact on students’ academic performances, and it is important for both educators and higher education policymakers to recognize this. In order to ensure a long-term improvement in academic performance, all stakeholders should recognize the value of social media as innovative and effective learning tools and as new methods of teaching and learning in virtual environments, which are constantly being sought out by educators. The value of these social media websites becomes increasingly important with the absence of formal learning management systems in many developing countries. They can be adopted as an educational tool and as a substitute to learning management systems to supplement conventional learning in the classroom.

## 7. Conclusions

COVID-19 raised the value of online learning for higher education institutions. Online learning in higher education is likely to undergo further changes post COVID-19, with the rapid acceleration of digital technology in learning and teaching. This study is one of many current attempts examining the value of online learning amid COVID-19 in developing countries, with a particular focus on the use of social media for e-learning in India. As a result of the COVID-19 pandemic, it has become clear that virtual learning is essential for the present and the future. A post-pandemic educational paradigm shift will require more than just infrastructure improvements. It is possible that this paradigm represents a shift away from traditional teaching methods such as lectures and group activities toward more student-centered methods, such as discussions and hands-on activities. The present is an opportune moment to examine and analyze the theoretical benefits of social media and consider their relative advantages for education through technology’s ability to improve student learning. Using social media is seen as critical for modern competency by both students and educators alike. The use of social media in educational contexts can promote learning, increase participation and engagement, disseminate content well and improve pedagogy. Additionally, social media websites could be a way for students to build social networks with other students of the same level to support each other globally. It could partially be adopted within the conventional classroom to enhance learning outcomes.

## 8. Limitation of the Study and Further Research

This study was concerned with Indian students’ perspectives of social media usage for e-learning amid COVID-19, their motivations for social media usage, and the impact of social media usage on their grades. The study was limited to a small sample of higher education students (most of them were postgraduate). The study used an online survey for data collection. Further research could be undertaken with a wider research sample and within different countries. Other tools of data collection could be adopted, such as focus groups with students to gain a deeper perspective about their perceptions of social media usage in future education, post COVID-19.

## Figures and Tables

**Table 1:** The profile of respondents and time spent on social media daily.

Profile		Frequency	%	
**Gender**				
Male		313	73	
Female		118	27	
**Education**				
Undergraduate		153	35	
Post Graduate		278	65	
**Subjects/Stream**				
Science/Engineering		154	36	
Arts/Social Science		179	41	
Commerce/management		98	23	
**Time Spent on social media on daily basis**				
**Time spent**	**Frequency**	**Percentage**	**Value (df)**	***p* value**
<1 h	42	10	25.189 (3)	0.000
1–2 h	215	50		
3–4 h	130	30		
>5 h	44	10		

**Table 2:** Social media platforms of choice.

Gender	YouTube	%	Facebook	%	Research Gate	%	Twitter	%	Others	%
Male	107	34	89	28	34	10	51	16	32	10
Female	47	39	34	28	22	18	9	7	6	5

**Table 3:** Motivations for using social media.

Purpose		Entertainment	Personal Use	Altruism	Information Seeking	Value (df)	*p* Value
**Gender**	Male	90	146	14	63	12.070 (3)	0.007
	Female	44	38	13	23		

**Table 4:** Impact on students’ grades.

Gender	Helped My Grade	Did Not Help My Grade
Male	240 (77%)	73 (23%)
Female	71 (60%)	47 (40%)

**Table 5:** Impact on overall academic performance.

Gender	Impact on Overall Academic Performance			Value (df)	*p* Value
	Highly Impact (Positive)	Moderate Impact	Highly Impact (Negative)		
Male	197 (63%)	79 (25%)	37 (11%)	27.064 (2)	0.000
Female	53 (45%)	26 (22%)	39 (33%)		

**Table 6:** Attitudes toward and beliefs about social media use in education.

Factors	Strongly Disagree	Disagree	Neither Agree Nor Disagree	Agree	Strongly Agree
Enjoy using social networking sites	15 (3.48)	18 (4.18)	31 (7.19)	153 (35.5)	214 (49.65)
Ease of use	11 (2.55)	13 (3.02)	47 (10.9)	159 (36.89)	201(46.64)
Better learning opportunities with social networking	7 (1.62)	4 (0.93)	22 (5.1)	195 (45.24)	203 (47.1)
Enjoy using social networking for assignments	84 (19.49)	69 (16.01)	78 (18.1)	98 (22.74)	102 (23.67)
Social networking is good for socializing not for learning	178 (41.3)	104 (24.13)	67 (15.55)	34 (7.89)	48 (11.14)
Social networking makes learning interactive/interaction with peers	22 (5.1)	16 (3.71)	38(8.82)	160 (37.12)	195 (45.24)
Social networking improving student outcomes	11 (2.55)	7 (1.62)	25 (5.8)	141 (32.71)	247 (57.31)
Social networking encourages sharing	76 (17.63)	49 (11.37)	118 (27.38)	91 (21.11)	97 (22.51)
Satisfied with social networking in collaborative learning environments	45 (10.44)	17 (3.94)	19 (4.41)	154 (35.73)	196 (45.48)
Using social networking is distracting in education	194 (45.01)	112 (25.99)	58 (13.46)	43 (9.98)	24 (5.57)

## Data Availability

Data is available upon request from researchers who meet the eligibility criteria. Kindly contact the first author privately through the e-mail.

## Appendix A

Dear Student,

We are conducting a study on the use of social media during COVID 19 for online learning purposes. We look for your valuable response based on the questions given below. This questionnaire is framed to collect data from the Post-Graduate and Undergraduate Students from different Indian colleges and universities.

Your response will be kept confidential and used only for this study purpose. Therefore, our kind requests you to please spare few minutes to fill up the questionnaire. Your cooperation in this regard will be very appreciable.

Best regards

The researchers

Note: please tick the most appropriate answer form your perspective.

**Section A**					
1. **Name (optional): ……………………**	**Contact: (optional): …………………**				
2. **Gender:**	Male Female				
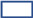
3. **Educational Qualification:**	Undergraduate Postgraduate				
4. **University/College Name (optional): ……………….**					
5. **Subject/Stream:**					
- Science/Engineering - Arts/Social Science - Commerce/management					
6. **Do you use social media for educational-related purposes?**					
YES NO *(Note: If **NO** SKIP all the questions below).*					
7. **How much Time you Spent on social media on daily basis in general?**					
- <1 h - 1–2 h - 3–4 h - >5 h					
**Section B**					
8. **Highlight the Social media platforms of your choice:**					
- YouTube - Facebook - Research Gate - Twitter - Others					
9. **What Motivates you to Use social media**					
- Entertainment - Personal Use - Altruism - Information seeking					
**Section C**					
10. **What is the Impact on your grade while you use social media?**					
- Helped my grade - Not helped my grade					
11. **How has Social Media use impacted your Overall Academic Performance?**					
- Highly impact (positive) - Moderate impact - Highly impact (Negative)					
12. **Attitudes toward and beliefs about social media use in education**					
**STATEMENTS**	**Strongly** **Disagree**	**Disagree**	**Neither Agree** **Nor Disagree**	**Agree**	**Strongly** **Agree**
Enjoy using social networking sites					
Ease of use					
Better learning opportunities with social networking					
Enjoy using social networking for assignments					
Social networking is good for socializing not for learning					
Social networking makes learning interactive/interaction with peers					
Social networking improving student outcomes					
Social networking encourages sharing					
Satisfied with social networking in collaborative learning environments					
Using social networking is distracting in education					

                     *****THANK YOU*****.
